# Short-term chloral hydrate as an add-on treatment may improve sleep and alleviate agitation in inpatients with treatment resistant schizophrenia: a retrospective case series study

**DOI:** 10.3389/fpsyt.2024.1293676

**Published:** 2024-02-29

**Authors:** Assaf Shelef, Habashi Alaa, Esther Bloemhof-Bris, Dania Halperin, Shira Weizman, Rafael Stryjer

**Affiliations:** ^1^ Lev Hasharon Mental Health Center, Tzur Moshe, Israel; ^2^ Faculty of Medicine, Tel Aviv University, Tel Aviv, Israel; ^3^ Abarbanel Mental Health Center, Bat Yam, Israel

**Keywords:** chloral hydrate, treatment resistant schizophrenia (TRS), sleep, violence, agitation

## Abstract

**Introduction:**

Chloral hydrate (CH), a medication dating back to 1832, is tranquilizer and sleep promoter still used today. It remains an option for short-term insomnia therapy and sedation before medical procedures, despite its controversial safety profile.

**Methods:**

This study investigated the potential benefits of chloral hydrate addition for increasing sleep duration and reducing agitation and violence in inpatients with treatment-resistant schizophrenia (TRS). A retrospective, observational case series design was utilized, analyzing data from fourteen patients diagnosed with TRS disorders.

**Results:**

CH addition increased the rate of full night sleep and decreased the rates of agitation and verbal and physical violence events. Notably, no adverse events including falls were reported during CH addition.

**Discussion:**

CH shows some short-term benefits in improving sleep disorders and reducing violent and agitated behavior in patients with TRS. Our study has limitations due to its small sample size, retrospective design and lack of a control group. A large-scale, double-blind, randomized trial is needed to further explore the efficacy and safety of CH in psychiatric populations with TRS accompanied by agitation, violence and disturbed sleep.

## Introduction

Chloral hydrate (CH) is a medication developed in 1832 by Justus von Liebig (1). Its main use is as sedative and hypnotic medication. Early in the 20th century, evidence showed that regular CH use could result in addiction (2, 3). Consequently, benzodiazepines were introduced as a replacement for CH (4).

CH is readily absorbed by the digestive system and rapidly metabolized by the liver into the metabolite trichloroethanol, which has a duration of action lasting eight to eleven hours in adults. Through its interactions with GABA-A and AMPA receptors, this drug acts as an antagonist to ion channels and calcium flow, ultimately suppressing the central nervous system (5).

The safety of CH is considered relatively high when used in the correct dosage, making it suitable for use in children as a pre-medication drug before medical procedures such as echocardiograph (6). CH is also used for management of chronic insomnia in elderly persons with a recommended dosage for sleep disorders of 500-1000mg (7, 8).

There are currently no FDA-approved drug products that contain CH in the USA, however CH is an approved product currently in use in Australia, Canada, Hong Kong and the UK. CH administration carries the risk of respirational depression and may also result in prolonged sedation, ventricular dysrhythmias, severe hypotension and irritating gastric effects (9, 10). The main side effects are digestive, cardiologic (risk of rhythm disorder), dermatologic, neuropsychiatric (withdrawn, delusions, hallucination, dependence) and ophthalmologic. Death could occur after absorption of overdoses of around 10 g of chloral hydrate (5).

According to the product monograph, reviewed by the Odan Laboratories in 2022, CH can lead to abuse, misuse, addiction, physical dependence and withdrawal reaction (11). Nowadays, CH is prescribed as a hypnotic drug with very limited use in psychiatry, mainly due to the widespread availability of benzodiazepines, which have undergone extensive research and have a better safety profile. CH 500mg has been found effective in inducing and maintaining sleep in a small sample of elderly inpatients with mild dementia (12). The authors were unable to find any studies on the efficacy of CH for inducing sleep, in patients with treatment resistant schizophrenia (TRS). The present study investigated the short-term (10 days) efficacy and safety of CH in patients with TRS.

## Methods

### Participants

This retrospective observational case series study was conducted in a regional mental health center in Israel between October 1^st^, 2021 and March 31^st^, 2022. The study protocol was approved by the local Institutional Review Board.

Data were extracted from the medical records of 14 inpatients (13 men and 1 woman) aged 32-60 years (µ 47.36, SD ± 10.19). The study population consisted of chronic inpatients diagnosed with TRS, hospitalized in a closed ward for at least six months. The patients received 1000 mg. CH oral solution, once a day, 30 minutes before bedtime, as recommended in the Chloral Hydrate Summary Report (13). The reason for CH add-on on those specific difficult patients was the need to find a solution for their sleep difficulties.

### Measures

Demographic and clinical data were extracted from the medical records for the day CH was started and included age, gender, main psychiatric diagnosis, comorbidities, previous hospitalizations and medications such as antipsychotics, mood stabilizers, and benzodiazepines.

The extracted clinical data were for the ten days prior to the CH treatment initiation and the ten days following its commencement. These data included: number of nights with uninterrupted sleep, of instances of isolation or restriction, of agitation events, of violence (verbal and physical), of reported adverse events (such as headaches or changes in blood pressure), of falls and of referrals to a general hospital.

### Statistical analyses

Due to the abnormal distribution of the data, Related-Samples Wilcoxon Signed Rank Tests were utilized for the analysis. All results are expressed as mean ± SD, median (Q1, Q3), or ratios (%) as appropriate. Statistical analyses were performed using SPSS ver. 28.0 (SPSS Inc., Armonk, NY). Statistical significance was set at p <0.05.

## Results

**Table 1** displays the participants’ demographic and clinical data.

**Table 1:** Demographic and Clinical Data of the Study Population (N = 14).

Normally distributed variables		Mean (±SD)
Age		47.36 (±10.19)
Number of typical antipsychotics		1.5 (±1.09)
Abnormally distributed variables		Median (Q1, Q3)
Number of previous hospitalizations		12 (4.5, 26.25)
Number of antipsychotics		2.5 (2, 3)
Number of atypical antipsychotics		1 (0, 2)
Categorical variables		N (%)
Gender	M	13 (93%)
	W	1 (7%)
Comorbidity	Yes	12 (86%)
	No	2 (14%)
Clozapine	Yes	6 (43%)
	No	8 (57%)
Benzodiazepines	Yes	12 (86%)
	No	2 (14%)
Mood stabilizers / Antiepileptic	Yes	6 (43%)
	No	8 (57%)

During the initial ten days of CH treatment, a statistically significant increase in the rate of nights with uninterrupted sleep was observed compared to the ten days before treatment.

Verbal and physical violence showed a significant decrease (**Table 2**).

**Table 2:** Comparison between the 10 Days Prior to Initiation of Chloral Hydrate Treatment and during 10 Days of Chloral Hydrate Addition.

Incident type	10 days prior to CH addition [Median (Q1, Q3)]	10 days of CH addition [Median (Q1, Q3)]	*P*
Uninterrupted night's sleep	9 (4,10)	10 (10,10)	0.018
Agitation	6 (2.75,10)	3.5 (0.75,7.25)	0.002
Verbal violence	4 (2,10)	2.5 (0.75,8)	0.011
Physical violence	0.5 (0,1.25)	0 (0,0.25)	0.011
Adverse events	0 (0,2)	0 (0,0)	NS
Falls	0	0	NS
Isolation and restriction	0 (0,1)	0 (0,0)	0.059
Emergency room referrals	0	0	NS

CH, Cloral Hydrate. NS, non-significant.

Adverse events, as abnormal laboratory results, falls or emergency room referrals, did not differ after ten days of CH treatment, compared to the then days before treatment (**Table 2**).

No significant differences were found in other variables.

## Discussion

Our study aimed to investigate whether CH as an add-on therapy for TRS inpatients improves sleep quality, agitation, and violence.

The population of inpatients with TRS often experiences disrupted sleep patterns, possibly linked to disturbances in their circadian rhythm. The authors were unable to find a treatment that has been developed specifically to address sleep disturbances in schizophrenia patients. When necessary, cognitive-behavioral therapy for insomnia is the preferred treatment choice (14). However, TRS inpatients tend to be affected by severe cognitive impairments with difficulties to express themselves, rendering cognitive-behavioral therapy challenging and difficult.

Our findings revealed significant improvements in night’s sleep, along with a notable decrease in agitation and in verbal and physical violence, following the initiation of CH treatment. Sleep disturbances in schizophrenia have been associated with dysregulation in calcium channel activity and alterations in GABA and glutamate neurotransmission (15). The interaction of CH with GABA receptors (16) may contribute to its beneficial effect on sleep quality. Agonistic modulators and agonists of GABA receptors are known to be positively involved in the induction and consolidation of NREM sleep (17). CH might have the same agonist effect on sleep regulation. No specific recent studies have been found in humans on the possible effect of CH on GABA receptors resulting in sleep improvement.

Although various side effects of CH have been reported and its safety questioned, as this drug toxic profile might be lethal at 4 to 10 times (4-10g) the recommended dosage (18, 19), we did not encounter any adverse events as abnormal laboratory results or falls in our study. Our results show that CH might be safe when used at the right dosage for inpatients suffering from TRS.

In conclusion, CH shows some short-term benefits in improving sleep disorders and reducing violent and agitated behavior in patients with TRS.

Our study was constrained, however, by its small sample size, lack of control group, selection bias and retrospective open-trial design. We suggest conducting a large-scale, double-blind, randomized trial to gain a more comprehensive understanding of CH’s efficacy and safety in psychiatric populations with severe mental illnesses. Such research would provide more robust evidence and valuable insights into the potential benefits of CH as a treatment option in TRS.

## Data availability statement

The raw data supporting the conclusions of this article will be made available by the authors, without undue reservation.

## Ethics statement

The studies involving humans were approved by Abarbanel Institutional Review Board. The studies were conducted in accordance with the local legislation and institutional requirements. Due to the retrospective study design, the study was approved as exempt from the need for written informed consent by the Abarbanel Institutional Review Board.

## Author contributions

AS: Methodology, Supervision, Writing – original draft. HA: Investigation, Methodology, Writing – original draft. EB: Formal Analysis, Methodology, Project administration, Software, Supervision, Writing – original draft. DH: Writing – review & editing. SW: Writing – review & editing. RS: Conceptualization, Investigation, Methodology, Project administration, Supervision, Validation, Writing – original draft.

## References

[1] Liebig J. Ueber die verbindungen, welche durch die einwirkung des chlors auf alkohol, aether, ölbildendes gas und essiggeist entstehen. Annalen Der Physik. (1832) 100(2):243–95. doi: 10.1002/andp.18321000206

[2] Chopra R Chopra GS. Chloral hydrate and paraldehyde as drugs of addiction. Indian Med Gazette. (1932) 67(9):481. PMC5231651 29010959

[3] Robinson J. A case of chloral hydrate addiction. Int J Soc Psychiatry. (1966) 12(1):66–71. doi: 10.1177/002076406601200110 5906147

[4] Tariq SH Pulisetty S. Pharmacotherapy for insomnia. Clinics Geriatric Med. (2008) 24(1):93–105. doi: 10.1016/j.cger.2007.08.009 18035234

[5] Gauillard J Cheref S Vacherontrystram MN Martin JC. Chloral hydrate: a hypnotic best forgotten? L'encephale. (2002) 28(3 Pt 1):200–4. 12091779

[6] Napoli KL Ingall CG Martin GR. Safety and efficacy of chloral hydrate sedation in children undergoing echocardiography. J Pediatr. (1996) 129(2):287–91. doi: 10.1016/S0022-3476(96)70256-1 8765629

[7] Bain KT. Management of chronic insomnia in elderly persons. Am J Geriatric Pharmacother. (2006) 4(2):168–92. doi: 10.1016/j.amjopharm.2006.06.006 16860264

[8] National Institute of Diabetes and Digestive and Kidney Diseases (US). LiverTox: clinical and research information on drug-induced liver injury. Natl Institute Diabetes Digestive Kidney Dis. (2012).

[9] Bowyer K Glasser SP. Chloral hydrate overdose and cardiac arrhythmias. Chest. (1980) 77(2):232–5. doi: 10.1378/chest.77.2.232 7353427

[10] Dallman JA Ignelzi MA Jr Briskie DM. Comparing the safety, efficacy and recovery of intransal midazolam vs. oral chloral hydrate and promethazine. Pediatr Dent. (2001) 23(5):424–30. 11699169

[11] Odan Laboratories. Product monograph including patient medication information: Chloral Hydrate Syrup Odan. (2022).

[12] Linnoila M Viukari M Numminen A Auvinen J. Efficacy and side effects of chloral hydrate and tryptophan as sleeping aids in psychogeriatric patients. Int Pharmacopsychiatry. (1980) 15(2):124–8. doi: 10.1159/000468423 7002832

[13] Yuen MV Gianturco SL Pavlech LL Storm KD Yoon S Mattingly AN. Chloral Hydrate: Summary Report. (2020).

[14] Trauer JM Qian MY Doyle JS Rajaratnam SM Cunnington D. Cognitive behavioral therapy for chronic insomnia: a systematic review and meta-analysis. Ann Internal Med. (2015) 163(3):191–204. doi: 10.7326/M14-2841 26054060

[15] Ferrarelli F. Sleep abnormalities in schizophrenia: state of the ar and next steps. Am J Psychiatry. (2021) 178(9):903–13. doi: 10.1176/appi.ajp.2020.20070968 PMC8446088 33726524

[16] Peoples RW Weight FF. Trichloroethanol potentiation of gamma-aminobutyric acid-activated chloride current in mouse hippocampal neurones. Br J Pharmacol. (1994) 113(2):555. doi: 10.1111/j.1476-5381.1994.tb17025.x 7834208 PMC1510122

[17] Lancel M. Role of GABAA receptors in the regulation of sleep: initial sleep responses to peripherally administered modulators and agonists. Sleep. (1999) 22(1):33–42. doi: 10.1093/sleep/22.1.33 9989364

[18] Steinberg AD. Should chloral hydrate be banned? Pediatrics. (1993) 92(3):442–6. 8361800

[19] Gerretsen M De Groot G van Heijst AN Maes RA. Chloral hydrate poisoning: its mechanism and therapy. Veterinary Hum Toxicol. (1979) 21:53–6. 505984

